# *PLoS Biology* in Action

**DOI:** 10.1371/journal.pbio.0020025

**Published:** 2004-01-20

**Authors:** Barbara Cohen

## Abstract

Some examples of how the *PLoS Biology* content is used and a request for feedback from creative users

In the first month after our launch, the PDF of the “monkey-robot” article by Miguel Nicolelis received tens of thousands of downloads. We are not sure who downloaded the paper because we do not ask people to register at our site. We suspect, however, that its popularity is in part due to the widespread media coverage of the article (from *Time* magazine to Al-Jazeera and *Die Zeit*), which demonstrates a thirst for the original scientific paper and makes a strong argument for open access. We were even more surprised by the popularity of the “issue PDF,” a whopping 72 MB file that contains the entire journal, cover to cover. The inaugural issue PDF was downloaded thousands of times within the first few weeks, even though we had printed 30,000 hard copies and distributed them widely.

## The Sky's the Limit (with Proper Attribution)

But who are our users, and what do they do with the content? We know that *PLoS Biology* articles are used in a variety of educational settings. For example, the Nicolelis article has already been used for several high-school science projects and by a psychology student who compared the original research paper to its media derivatives. And the paper by Joseph DeRisi and colleagues on the malaria transcriptome has served as the basis for a continuing medical education drug discovery class and has been the topic of several undergraduate classes.

Under the terms of the Creative Commons Attribution License, not only can *PLoS Biology* articles be reproduced and distributed without the need to obtain explicit permission, they can also be used for the publication of derivative works. Two *PLoS Biology* articles have already been entered in the *Internet Encyclopedia*. The source of the articles is clearly cited; it is also clear that they have been modified ed by the addition of extra links and information and that they are editable by users of the encyclopedia. Although this is an experiment in freely available and editable information, there will also be opportunities for entrepreneurs to produce derivative works with the type of added value that some users might wish to pay for. Open access provides free access to the research literature, but also provides publishers with new commercial opportunities.

Once a significant body of full-text literature is available, it also becomes possible to use it for the development of new tools and resources for text- and data-mining and knowledge discovery. We plan to collaborate with developers of such tools. For their use—and for anybody else who likes their text “marked-up”—we make the XML version of our articles available. These are formatted according to the Journal Publishing DTD (Document Type Definition) from the United States government's National Library of Medicine, which provides a standard for archiving and exchanging XML versions of published documents.

## Overcoming Obstacles

A barrier for many potential users is that all our content—at least for now—is in English. We are delighted to hear that some of it is already being translated into other languages for local use. The feature article on the environmental benefits and risks of genetically modified crops, for example, will be republished (in Spanish) in the Argentinian environmental magazine *Gerencia Ambiental*. We hope that this will catch on—and urge anyone translating our content to let us know so that we can point others to the various language versions. We merely ask that the translators and their publishers acknowledge the authors and the source, by including a statement such as “this is a translation from the original article by Virginia Gewin published in *PLoS Biology*, DOI: 10.1371/journal.pbio.0000008.” Almost any translation is better than none for those excluded by language barriers, but quality control is a concern, and we are keen to collaborate with individuals or organizations who are interested in providing high-quality translations for some or all of our content on a regular basis.

Internet connectivity is another obstacle. We know that some of the downloads of the issue PDF were transferred onto CD-ROMs that were copied and distributed in Uganda and Cambodia, areas in which Internet access is often slow and expensive. Other copies of the PDF were being used to create local hardcopies of the journal for communal use. We are happy to support these and related efforts to bring *PLoS Biology* content to readers by, for example, increasing the range of formats available at our Web site. Let us know what would help.

## Wanted: More Feedback

Besides the encouraging Web statistics, we have heard from many individual users since the launch of *PLoS Biology*. We'd like to hear from even more. Tell us how you use *PLoS Biology*: your ideas might inspire others. As a way of building on the work and ideas of others, we have added a page on the PLoS Web site (www.plos.org/creative_uses) where we list some of the more creative and unusual uses of *PLoS Biology*. Let us know what you do with *PLoS Biology*, or what you'd like to do, and we'll see what we can do to make it possible.
